# Domain-Specific Common Data Elements for Rare Disease Registration: Conceptual Approach of a European Joint Initiative Toward Semantic Interoperability in Rare Disease Research

**DOI:** 10.2196/32158

**Published:** 2022-05-20

**Authors:** Haitham Abaza, Dennis Kadioglu, Simona Martin, Andri Papadopoulou, Bruna dos Santos Vieira, Franz Schaefer, Holger Storf

**Affiliations:** 1 Institute of Medical Informatics Goethe University Frankfurt University Hospital Frankfurt Frankfurt am Main Germany; 2 European Commission Joint Research Centre Ispra Italy; 3 Department of Medical Imaging Radboud Institute for Health Sciences Radboud University Medical Center Nijmegen Netherlands; 4 Center for Molecular and Biomolecular Informatics Radboud Institute for Molecular Life Sciences Radboud University Medical Center Nijmegen Netherlands; 5 Division of Pediatric Nephrology Center for Pediatrics and Adolescent Medicine Heidelberg Germany

**Keywords:** semantic interoperability, common data elements, standardization, data collection, data discoverability, rare diseases, EJP RD, EU RD Platform, ERNs, FAIRification, health infrastructure, industry, medical informatics, health platforms, health registries, health and research platforms, health domains

## Abstract

**Background:**

With hundreds of registries across Europe, rare diseases (RDs) suffer from fragmented knowledge, expertise, and research. A joint initiative of the European Commission Joint Research Center and its European Platform on Rare Disease Registration (EU RD Platform), the European Reference Networks (ERNs), and the European Joint Programme on Rare Diseases (EJP RD) was launched in 2020. The purpose was to extend the set of common data elements (CDEs) for RD registration by defining domain-specific CDEs (DCDEs).

**Objective:**

This study aims to introduce and assess the feasibility of the concept of a joint initiative that unites the efforts of the European Platform on Rare Disease Registration Platform, ERNs, and European Joint Programme on Rare Diseases toward extending RD CDEs, aiming to improve the semantic interoperability of RD registries and enhance the quality of RD research.

**Methods:**

A joint conference was conducted in December 2020. All 24 ERNs were invited. Before the conference, a survey was communicated to all ERNs, proposing 18 medical domains and requesting them to identify highly relevant choices. After the conference, a 3-phase plan for defining and modeling DCDEs was drafted. Expected outcomes included harmonized lists of DCDEs.

**Results:**

All ERNs attended the conference. The survey results indicated that genetic, congenital, pediatric, and cancer were the most overlapping domains. Accordingly, the proposed list was reorganized into 10 domain groups and recommunicated to all ERNs, aiming at a smaller number of domains.

**Conclusions:**

The approach described for defining DCDEs appears to be feasible. However, it remains dynamic and should be repeated regularly based on arising research needs.

## Introduction

### Background

Patient registries and databases are fundamental instruments for increasing knowledge on rare diseases (RDs), supporting clinical, epidemiological, and basic research, and improving patient care and health care planning [1,2]. With >600 registries across Europe [3], RDs suffer from fragmented knowledge, scattered expertise, and research duplication [1]. Data are not collected in a uniform way throughout Europe, and there are no shared standards to analyze the information [3]. The use of international coding and nomenclature, minimal common data sets, and good practice guidelines enhances the interoperability and maximizes the utility of RD registries. This allows data to be efficiently pooled to reach sufficient sample sizes for clinical and public health research focusing on disease etiology, pathogenesis, diagnosis, and therapy [1,2].

On the Rare Disease Day (2019), the European Commission announced a new web-based knowledge-sharing platform to promote better diagnosis and treatment for >30 million patients with RD. Developed by the Joint Research Centre (JRC) of the European Commission, the EU RD Platform aims to bring together European RD registries, thus overcoming fragmentation and promoting interoperability between existing and new registries. Moreover, the platform seeks to standardize data collection and data exchange at the EU level, thereby supporting quality RD research, enhancing diagnosis and treatment outcomes, and improving the lives of patients and their families [4]. The efforts of European Reference Networks (ERNs) toward establishing ERN-wide registries are also implicitly supported by the platform [3], mainly by offering a patient pseudonymization and privacy-preserving linkage service.

By delivering EU standards for data collection and data sharing, the EU RD Platform is a significant asset for the European Joint Programme on Rare Diseases (EJP RD) [5], which aims to establish an innovation network for rapidly translating research results into clinical and health care applications [3]. The EJP RD brings together over 130 institutions from 35 countries to collaboratively build infrastructure and digital platforms, which promote cross-border sharing of clinical data and expertise. The ultimate goal is to overcome the fragmentation of RD resources and to foster RD care and medical innovation. The EJP RD also aims to use, support, and connect already-funded tools operating within the field of RD research and adapt them to the needs of end users through implementation tests in real settings [6]. Through EJP RD, the EU RD Platform resources can be disseminated to future research projects and exposed to a wider community of RD researchers, clinicians, and patients in Europe and elsewhere [3].

Aiming to make RD registries and their data searchable and findable, the EU RD Platform comprises the European Rare Disease Registry Infrastructure (ERDRI) [7], which includes the European Directory of Registries (ERDRI.dor), the Central Metadata Repository (ERDRI.mdr), and the pseudonymization tool. Details on their infrastructure and functioning will be published elsewhere. However, we focus here on the set of common data elements (CDEs) for RD registration [8], which is another important building block of the platform. Developed by experts from various EU projects (eg, European Union Committee of Experts on Rare Diseases Joint Action [EUCERD], European Platform for Rare Disease Registries [EPIRARE], and RD-Connect) related to common data sets, the set of CDEs was released by the EU RD Platform as the first practical instrument toward increasing the interoperability of RD registries [9]. The set recommends the collection of 16 data elements by all European RD registries, as they are considered essential for RD research. The 16 CDEs are classified into various groups, including personal data, diagnosis, disease history, care pathway, information for research purposes, and a disability profile. Exemplary CDEs include age (date of birth), sex (male, female, undetermined, or fetus), status (alive, dead, lost to follow-up, or opted-out), and RD diagnosis (ORPHAcode) [9].

Although CDEs constitute a common basis for characterizing patients with RD across all 24 ERNs, many overlaps between ERN domains are not clearly defined. For instance, there are 3 oncological (ERN PaedCan, ERN EURACAN, and ERN EuroBloodNet) and 3 neurological (ERN EpiCare, ERN-RND, and ERN EURO-NMD) ERNs, among others, with numerous diseases covered by each of them being treated jointly. The list of the 24 initially funded ERNs that have been considered in the context of this work can be found in Multimedia Appendix 1. Furthermore, beyond CDEs, many ERN registries collect data elements that may be commonly used by others working in the same domain. However, no standards exist for categorizing such commonalities. Domain-specific CDEs (DCDEs) are designed for use in studies or registries of a particular topic, disease or condition, body system, or other classifications (eg, cancer, Parkinson disease, Alzheimer disease, diabetes, or ophthalmology). Some domains are broadly applicable to a wide range of studies, whereas others are more useful in specific fields of clinical research [10]. Therefore, the definition of DCDEs for the various RD domains is expected to standardize data collection, thus enhancing the interoperability and facilitating the discoverability of data stored in RD registries.

In 2019, the EJP RD formed an expert workforce to assist the ERN Registry Task Force (TF) on interoperability and standardization issues [11,12]. Extracted from the data dictionaries of the first 4 ERN registries (ERKReg [ERKNet], U-IMD [MetabERN], EURRECA [ENDO-ERN], and DATA WAREHOUSE [ERN-LUNG]), a Common Data Dictionary (CDD) was introduced as a tool to avoid fragmentation and ensure registry interoperability. Accordingly, the TF committed to the use of the CDD as part of the group’s efforts to develop ERN registries in full compliance with the FAIR principles (findability, accessibility, interoperability, and reusability). Therefore, these efforts are expected to improve research transparency and facilitate knowledge discovery for both humans and machines [11,13].

### Rationale

To achieve semantic interoperability between RD registries, a joint initiative of the EU RD Platform, the ERN Registry TF, and the EJP RD registry interoperability work focus group was launched in 2020. Driven by the research needs of ERNs, the purpose was to extend the set of CDEs for RD registration by defining DCDEs, that is, ones that are considered necessary within each particular ERN domain. This idea was first expressed at an ERN Registry TF meeting in Brussels around mid-2019, when some ERNs indicated that they had already collected a small number of data elements that commonly exist in registries of their domain.

To this end, a joint conference took place in December 2020, bringing together the EU RD Platform team members, ERN representatives and registry owners, and EJP RD partners to discuss the concept of DCDEs cooperatively. The conference also aimed to tackle core questions, such as whether all ERNs already had a defined data set in place, and for which reasons they believed DCDEs would be necessary. Medical experts were also intended to indicate, during and after the conference, whether some domains could already be derived from existing overlaps and consider experts who could take charge of such domains. Exploring how harmonized lists of DCDEs could be produced was planned at a later stage, as well as identifying appropriate standards, ontologies, and terminologies to finally annotate DCDEs and integrate them into CDE semantic modeling activities of EJP RD.

Led by the ERN Registry TF, initial efforts were already made by 4 ERNs (ERKNet, MetabERN, ENDO-ERN, and ERN-LUNG) toward the creation of a CDD, mainly collecting common data fields among their registries in an Excel (Microsoft Inc) table. In continuation to these efforts, the plan is to have medical experts from all 24 ERNs drive this initiative toward the following:

- Forming ERN domain groups
- Defining DCDEs for each domain group by identifying commonalities among relevant ERNs
- Harmonizing, modeling, and publishing DCDEs and adding them to ERDRI.mdr

### Objectives

This study introduces the concept of a collaborative initiative, which aims to prevent duplicated efforts by uniting and coordinating the activities of the EU RD Platform, ERN Registry TF, and EJP RD on topics such as the CDEs, CDD, and common metadata and data model of the EJP RD. Moreover, the initiative aims to further standardize RD registration by extending the set of CDEs with DCDEs, thereby improving the semantic interoperability of RD registries and enhancing the quality of RD research. The study also assesses the feasibility of the concept by examining previous efforts to define (D)CDEs and exploring if and how DCDEs can benefit the RD field from the perspective of the ERNs.

## Methods

### Conference Participants

All 24 ERNs were invited to attend the conference. Speakers included participants from the JRC/EU RD Platform as well as EJP RD experts from various backgrounds, particularly focusing on common data sets and FAIRification. ERN representatives were preferably required to have a medical background and considerable involvement in registry activities. These were considered necessary requirements to identify essential ERN domains as well as existing overlaps, if any, thus paving the way for creating lists of DCDEs. The EJP RD previously built a database of experts involved in registry design and construction, listing their names, institutions, contact details, and expertise. Although not yet complete, the database included experts from all 3 parties (JRC/EU RD Platform, ERNs, and EJP RD) who indicated working with registries. Therefore, it was intended for use, together with other resources, to identify appropriate participants for the next steps.

### Preconference Tasks

Before the conference, a Forms (Microsoft Inc) survey was prepared and communicated to all 24 ERNs through the FAIRification stewards of the EJP RD. The survey proposed a list of medical domains and requested ERNs to identify those that generally fit their activities. The list comprised 18 domains, mainly suggesting the specialties indicated in the name of each ERN (eg, ERN-EYE—Sight, ERKNet—Renal, and EURACAN—Cancer). The main survey item read, “To which domain(s) do you think your ERN fits?” and enabled checking multiple answers. Another optional item asked if any of the suggested domains could be grouped together and allowed for text answers (eg, cancer and congenital). The deadline for completing the survey was set on the day of the conference. However, we also planned to collect any missing answers during or shortly after the conference.

### The Conference

Organized by the EJP RD, the conference comprised three 40-minute sessions, the second of which was dedicated to DCDEs. The first presentation was held by the EU RD Platform team, providing some findings collected in preparation for their originally planned ERN workshop in March 2020. Unfortunately, this event was cancelled because of the COVID-19 pandemic. The team also expressed interest in having 2 specific questions answered, namely, whether each of the ERNs already had their data set in place (at the ERN level) and for which specific purpose they believed DCDEs were necessary. The survey results were then presented by EJP RD experts, giving some exemplary purposes to illustrate the importance of DCDEs and accordingly suggesting a scoring method for rating the importance of every identified DCDE within a particular domain. It was also indicated that a technical phase would follow the definition of DCDEs. Therefore, both EJP RD and EU RD Platform experts would guide the ERNs through harmonizing and modeling identified DCDEs, in preparation for adding them to ERDRI.mdr and extending semantic data modeling activities of the EJP RD.

### Postconference Tasks

Following the conference, the remaining ERNs, which had not completed the survey, were requested to provide their answers, and the following 3-phase plan was jointly drafted:

1. Formation of ERN domain groups
  EJP RD experts group suggested domains and request ERNs to review the groups
  EJP RD experts request ERNs to reorganize the suggested groups, if necessary, aiming for a minimum number of domain groups and a minimum number of ERNs per group
  ERNs elect relevant experts/curation team members for each group
2. Definition of DCDEs
  Elected ERN experts suggest, curate, and define DCDEs by comparing their data dictionaries and identifying relevant commonalities for every domain group (the EJP RD can support if the data dictionaries are shared)
  The EU RD Platform and EJP RD experts offer support by providing necessary templates for DCDE lists, scheduling domain meetings, and ensuring that everything is harmonized
  The EU RD Platform and EJP RD experts prioritize DCDEs using the scoring method proposed by EJP RD, in case long lists are identified
3. Technical phase
  EU RD Platform and EJP RD experts guide ERNs to extend the semantic data modeling activities of the EJP RD: harmonization, modeling, and mapping of DCDEs
  EU RD Platform and EJP RD experts guide the ERNs in publishing DCDEs alongside CDEs and inclusion in ERDRI.mdr
  New registries implement both CDEs and DCDEs

### Scoring Method

Completed DCDE lists were planned to be sent to medical experts for review using a structured feedback method, allowing them to rate an arranged set of statements designed to indicate the relevance of each DCDE within a certain domain group. To ease the adoption of identified DCDEs by all RD registries, it was initially recommended that the rating statements address the following aspects: (1) importance of each DCDE for the integrity of a registry within a certain domain group, (2) reliability of data collection in each DCDE, (3) necessity of a DCDE for the analysis of the primary outcome of the registry, and (4) the time and cost required to collect each DCDE [14]. Other categories that might arise in discussions during or after the conference were also to be incorporated. On the basis of the feedback of experts, individual scores could eventually be calculated for every identified DCDE, thus reflecting the importance of each DCDE within each domain group. These scores could also be used by curation teams to prioritize their DCDEs, if their efforts culminated in prolonged lists.

### Domain Representation

**Figure 1:**
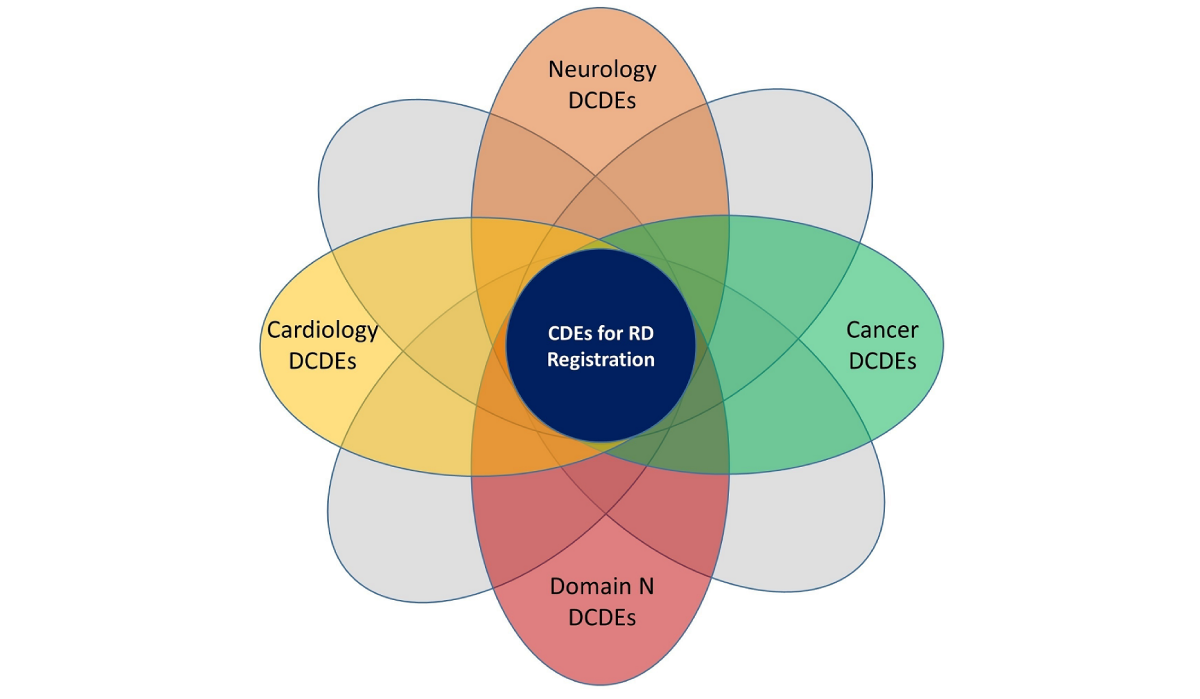
Domain-specific common data elements (DCDEs) classification and domain overlaps. CDE: common data element; RD: rare disease.

To visually represent the domains, several diagrams were prepared and circulated before and during the conference. Figure 1 illustrates this concept by showing domain overlaps and classifying DCDEs. Neurology, cancer, and cardiology were used for exemplary purposes, with *N* suggesting an unknown number of domains expected to be identified by the initiative. The CDEs of the EU RD Platform were placed in the center, ensuring that they would remain the basis for all DCDE lists.

**Figure 2:**
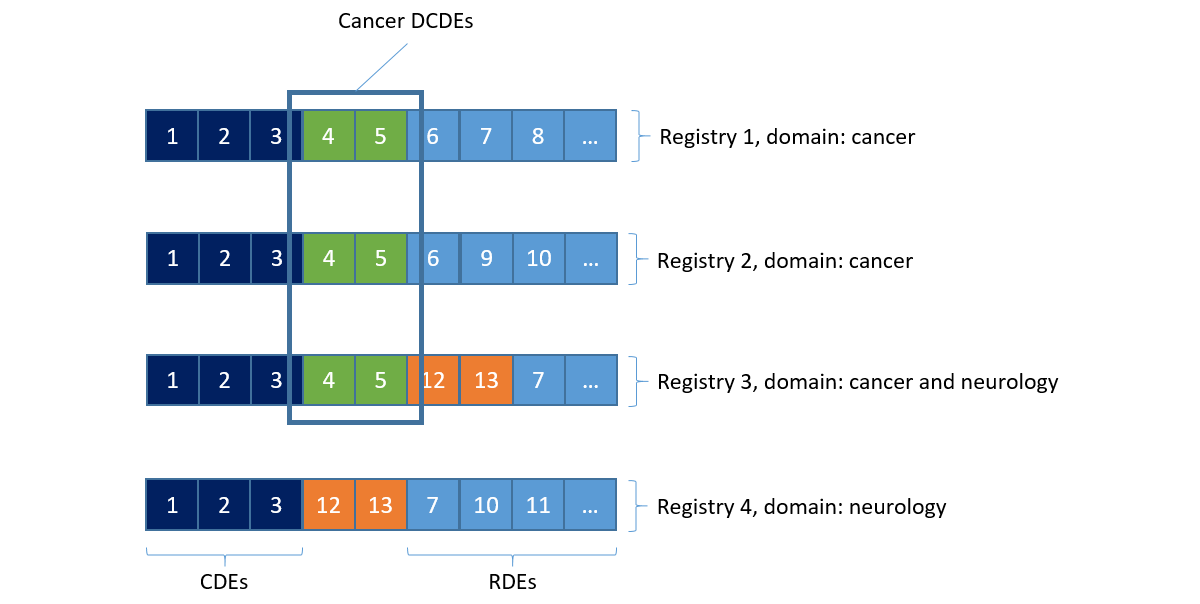
Classification of registries and their data elements. CDE: common data element; DCDE: domain-specific common data element; RDE: registry-specific data element.

To illustrate the concept further, and how it is intended to classify registries, Figure 2 depicts an example of 4 ERN registries belonging to 2 exemplary domains, namely cancer and neurology. Numbered blocks were used to represent the data elements of a registry, whereas colors were applied to characterize the different types of data elements. In an ideal world, the registry data elements could be classified into the following:

1. CDEs, that is, ones that would commonly exist in all RD registries
2. DCDEs, that is, ones that would commonly exist in all registries of a particular RD domain
3. Registry-specific data elements, that is, ones considered specific for each registry’s purpose, thus would commonly differ from one registry to another

This classification was also illustrated by the occurrence of navy blue data elements (CDEs) among all registries, regardless of their domain, as opposed to the green and orange elements present only in registries of the cancer and neurology domains, respectively. Furthermore, the presence of both the green and orange elements in registry 3 was used to indicate that a registry could belong to more than one domain, thereby incorporating DCDEs of both the cancer and neurology domains. Notably, the marked cancer DCDEs ensured that CDEs would constantly remain the basis for all domains while complemented by domain-specific extensions.

### Expected Outcomes

**Figure 3:**
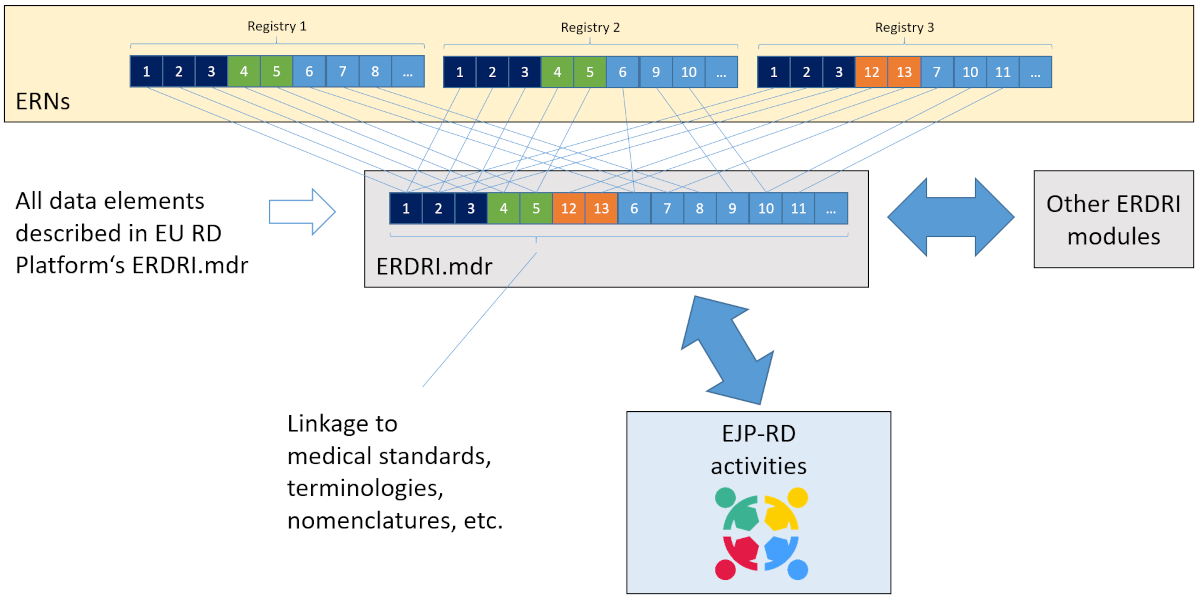
Technical vision. CDE: common data element; EJP RD: European Joint Programme on Rare Diseases; ERDRI: European Rare Disease Registry Infrastructure; ERN: European Reference Network; EU RD Platform: European Platform on Rare Disease Registration; MDR: Metadata Repository.

Lists of DCDEs for every identified ERN domain group were set as expected outcomes for the first and second phases. The technical phase was planned to harmonize these lists, thus removing duplicates, if any, and ensuring identical definitions of DCDEs and their values. The harmonized version would then be published in PDF format on the EU RD Platform and added to ERDRI.mdr to allow reusability. EJP RD experts would also explore existing ontologies and terminologies for annotating and incorporating the harmonized lists into the registry codebook of the EJP RD [15] and CDE semantic data model [16]. This is expected to enhance the interoperability of RD registries and boost RD research, which is the main objective of this project. Figure 3 shows a visualization of the concept, portraying the connections among the different members of the initiative.

### Ethics Approval

Any data examined do not relate to specific individuals, which means that no personal harm can occur to individuals. Accordingly, the review by an ethics committee was not required.

## Results

### Results Overview

All 24 ERNs attended the conference. Most participants indicated that they already had their data sets in place. However, the main purpose for defining DCDEs could not be recognized. The EJP RD experts, however, presented three potential purposes that emphasized the importance of DCDEs: increasing interoperability, allowing data comparisons in joint research projects, and improving data discoverability.

### A Scoring Method for the Nomination of Domain-Specific Common Data Elements

**Figure 4:**
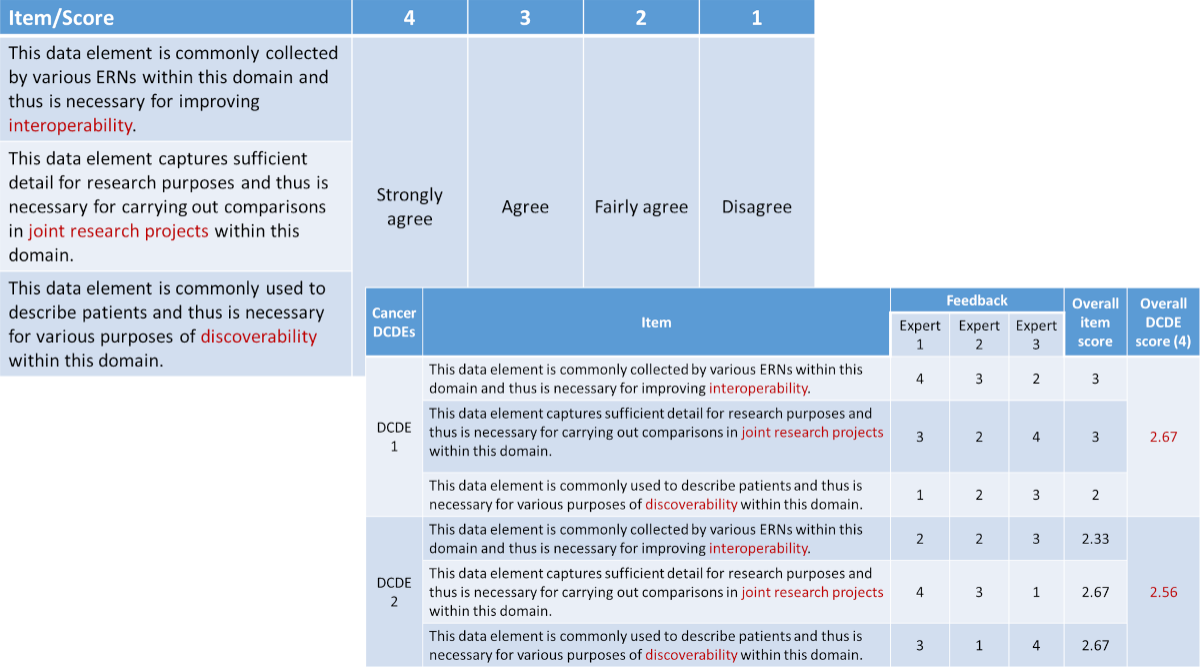
Proposed scoring system and example. DCDE: domain-specific common data element; ERN: European Reference Network.

A scoring method was also presented, suggesting a way for medical experts to rate the importance of each DCDE based on these 3 purposes. Figure 4 shows the proposed scoring system using a 4-point scale. On the basis of the feedback of 3 experts, exemplary scores were also provided for the 2 DCDEs belonging to the cancer domain. For each item, the average of all expert scores was calculated to provide an overall item score. The overall DCDE score was then determined as the average of all 3 overall item scores. In this particular example, the score of DCDE 1 slightly exceeded that of DCDE 2, suggesting that it is somewhat more important for the cancer domain. Similarly, individual tables are meant to be constructed for each of the domains identified by the initiative.

### First Proposal of Domains and Domain Groups

EJP RD experts also presented the survey results, indicating the genetic, congenital, pediatric, and cancer domains as the most overlapping and adding to the importance of defining DCDEs. For the first survey item, which requested ERNs to select relevant domains from a list of 18 suggestions, responses from 22 (92%) ERNs were received and are presented in Table 1. The second survey item was answered by 14 (58%) ERNs. However, no patterns could be identified in the suggested domain groups.

Following the conference, the EJP RD experts and FAIRification stewards grouped some of the suggested domains, aiming at a minimum number of domain groups. The initially proposed domains were reorganized into 10 domain groups, and the survey answers were used to identify relevant ERNs. Table 2 shows the suggested list of domain groups as well as the corresponding ERNs. As shown, a single domain group could comprise multiple related ERNs and a single ERN could belong to various relevant domain groups. The list was communicated once more to all ERNs, requesting them to edit, merge, add, or move their ERN between domains as they saw necessary. They were also requested to elect, for every domain group, a person in charge and members of a data element curation team. This constitutes selected members, from every ERN of a particular domain group, intended to be in charge of drafting a DCDEs list, sending it to medical experts for review using the aforementioned scoring method, and accordingly agreeing on final definitions.

**Table 1:** Survey responses.

Domain	ERNs^a^, n (%)
Genetic	20 (83)
Pediatrics	18 (75)
Congenital	14 (58)
Cancer	7 (29)
Endocrine and metabolism	6 (25)
Neurology	6 (25)
Renal	6 (25)
Immune disorders	6 (25)
Skin	5 (21)
Gastroenterology and hepatology	4 (17)
Lung	4 (17)
Other	4 (17)
Muscle, bone, and skeletal diseases	3 (13)
Cardiovascular	3 (13)
Hematological disorders	3 (13)
Urology	3 (13)
Respiratory	2 (8)
Head and neck	1 (4)
Sight	1 (4)

^a^ERN: European Reference Network.

**Table 2:** Suggested domain groups.

Domain group	Relevant ERNs^a^
Genetic	ERN-EYE, RARE-LIVER, ERNICA, ERKNet, ERN ITHACA, ERN GUARD-Heart, EPICARE, PaedCan, VASCERN, EuroBloodNet, Endo-ERN and ERN-BOND, MetabERN, ERN-TransplantChild, ERN-Skin, ERN CRANIO, ERN GENTURIS, RITA, and ERN Eurogen
Congenital	ERN-EYE, ERNICA, ERN ITHACA, VASCERN, Endo-ERN and ERN-BOND, MetabERN, ERN-TransplantChild, ERN-LUNG, ERN-Skin, ERN CRANIO, RITA, and ERN eUROGEN
Pediatrics	ERN-EYE, RARE-LIVER, ERNICA, ERN ITHACA, EPICARE, PaedCan, VASCERN, Endo-ERN and ERN-BOND, MetabERN, ERN-TransplantChild, ERN-LUNG, ERN-Skin, ERN CRANIO, ERN GENTURIS, RITA, ERN eUROGEN, and ERKNet
Cancer	RARE-LIVER, PaedCan ERN, ERN-EuroBloodNet, Endo-ERN and ERN-BOND, eUROGEN, ERN-Skin, and ERN GENTURIS
Neurological	ERKNet, EPICARE, ERN-RND, MetabERN, ITHACA, ERN Eurogen, and EURO-NMD
Immune and blood	RARE-LIVER, EPICARE, ERN-EuroBloodNet, ERN-TransplantChild, ERN-Skin, RITA, ERKNet, VASCERN, and ReConnet
Renal and urological	ERKNet, EPICARE, ERN-RND, Endo-ERN and ERN-BOND, MetabERN, ITHACA, ERN eUROGEN, ERN-EuroBloodNet, and ERN-TransplantChild
Respiratory and lung	VASCERN, ERN-EuroBloodNet, ERN-TransplantChild, ERN-LUNG, and ERNICA
Surgical	eUROGEN, ERN CRANIO, and ERN-TransplantChild
Muscle, bone, and skeletal	Endo-ERN and ERN-BOND, ITHACA, RITA, and EURO-NMD

^a^ERN: European Reference Network.

### Sustain the DCDE Implementation and Evolution

Upon receiving feedback from ERNs, the EJP RD and the EU RD Platform teams plan to offer support by organizing domain group meetings and providing templates for listing and describing DCDEs. In this sense, the EU RD Platform team formed a cancer working group, composed of cancer-related ERNs, to focus on identifying cancer DCDEs. The FAIRification stewards of the EJP RD also aim to ask all ERNs to share their data dictionaries to compare them and support the identification of commonalities within every domain group. Comparisons are meant to follow the approach and format of the existing CDD, previously performed for 4 ERNs and currently only listing CDEs. Following the conference, EJP RD also started organizing weekly web-based meetings (coffee rounds) as well as technical workshops, aiming to answer the ERNs’ frequently asked questions on several topics of interest. Relevant topics included the definition and use of CDEs, CDEs minimal data set, CDEs semantic model, and modeling DCDEs. The plan is to eventually expand the semantic model of the EJP RD with identified DCDEs, publish them on the EU RD Platform alongside CDEs, and add them to ERDRI.mdr.

## Discussion

### Overview

This paper presented a series of joint activities aiming to extend the EU RD Platform’s CDEs with DCDEs. The series starts with a strict medical phase, seeking to compile lists of DCDEs that commonly exist among registries of every ERN domain. A technical phase then follows in which a mix of medical and technical expertise primarily tackles harmonization and standardization issues. The results of each phase will be published separately. However, it is promising to review here, some of the previous efforts related to defining CDEs and identify connections to the current EU RD Platform, ERN, and EJP RD initiatives, if any.

### Previous Work

The term CDEs has been first introduced to the RD field by the US initiative National Institutes of Health/National Center for Advancing Translational Sciences Global Rare Diseases Patient Registry Data Repository Program [17]. Aiming at better data standardization and interoperability for RD registries, the program defined 75 database fields required for the establishment of any RD registry [18,19]. On the basis of these attributes, the RD-Connect and EPIRARE projects developed minimum data sets for patient data entry to be used in their own framework. They also encouraged continuous alignment with the Minimal Data Elements of the European Union Committee of Experts on Rare Diseases (EUCERD) Joint Action initiative, thereby improving cooperation among RD registries at the European level [17,20-22].

Although not strictly focused on RDs, the National Institute for Neurological Disorders and Stroke initiated the Common Data Elements Project in 2005, seeking to identify the core CDEs necessary for collection in all neuroscience clinical research studies [23]. To collect CDEs, the project used case report forms (CRFs) from various clinical studies and indicated that their work was dynamic and would continue to evolve over time based on arising needs. In addition to publishing core CDEs on their website [24], they continued to identify disease-specific CDEs using a 10-step process. Similar to what has been proposed for our joint activities, their steps involved a domain working group, a draft DCDEs list, and a review process. However, deeper steps toward data standardization were also involved. In addition to defining general DCDEs for the neurological domain, they have complemented those over the years with more specific DCDEs for diseases such as epilepsy, stroke, Parkinson disease, multiple sclerosis, and headache [23]. To date, the National Institute for Neurological Disorders and Stroke CDEs project has collected data standards for 24 neurological diseases and disorders [25].

Other efforts to define DCDEs have also been made by the National Cancer Institute, which sought to identify CDEs for cancer research, thereby facilitating data interchange and interoperability between cancer research centers [20,26]. DCDEs have also been collected in a joint initiative between the Radiological Society of North America and the American College of Radiology, producing a data dictionary of radiology CDEs for various domains. These included cardiac radiology, breast imaging, chest radiology, and head and neck imaging. The initiative aimed to foster the interoperability of data present in radiologic reports and images throughout different radiologic information systems, ultimately improving research and clinical practice [27,28]. The US National Library of Medicine has also compiled a repository of >20,000 data elements, seeking to improve data quality and facilitate data comparisons among various research studies. Furthermore, it aimed to allow for opportunities to compare and combine data from multiple studies with those stored in electronic health records [20,29].

In an effort to facilitate finding necessary expertise, as well as sufficient numbers of patients for RD research, the French national minimal data set has been introduced. After systematically reviewing the scientific literature on RD CDEs, 58 data elements were represented in the data set. These were considered the clinical data standard for all French RD centers as part of the French National Plan for Rare Diseases. The methodology used to identify the minimal data set adopted the Global Rare Diseases Patient Registry Data Repository CDEs as a gold standard and also implemented many common steps with our proposed approach. These included a first working group to put together an initial CDEs draft, submitting the draft to a panel of experts, and receiving validation via a survey instrument [20,30].

### Synergies With Other Activities Within the EJP RD

Our proposed approach could then be regarded as a continuation to previous efforts on DCDEs, seeking to expand the EU RD Platform’s CDEs standard at the European level. It also supports ongoing and future efforts in various areas within the EJP RD. For instance, it aligns with the project’s ERN-related activities, planning to hold 2 workshop series following the identification of DCDEs. The first series addresses various aspects of FAIRification, providing a set of discoverability metadata fields (metadata CDEs) that are considered the basis for describing resources and making them findable. The second series focuses on patient matchmaking, providing a means for querying scattered patient data sets to locate similar patients with RD, either within a single ERN or across multiple ERNs. In such workshops, having DCDEs would allow the identification of discoverability metadata to be focused on certain domains. Moreover, identified DCDEs, in addition to CDEs, would present the basic parameters for queries aimed at finding similar patients. This is also the focus of the Query Builder activities of the EJP RD, running 2 pilot projects on federated discovery of resources (eg, registries and biobanks) and record-level data (eg, patients and samples). However, details of these pilots are outside the scope of this paper and will be published elsewhere.

Our approach also integrates with FAIRification activities of the EJP RD, expanding the scope of the CDEs codebook and semantic model to include DCDEs, and facilitating data exchange among institutions that use different electronic data capture software. Together with an interoperable CRF generator tool [31], the codebook content could be used by all 24 ERNs to create and reuse interoperable CRFs, sparing the need to design new electronic CRFs while implementing their registries, at least for commonly used data elements. Therefore, by incorporating DCDEs, the codebook adheres to the EU RD Platform’s standard, requiring and enabling new registries to include both CDEs and DCDEs. Our efforts to define DCDEs could also take the ERN Registry TF’s initiative toward a CDD further, leading to an updated version that includes DCDEs in addition to CDEs, ensuring it is harmonized among participating ERNs, and extending it to all 24 ERNs.

### Conclusions

This paper presented a joint initiative of the ERNs, EU RD Platform, and EJP RD, aiming to define DCDEs for RD registration. The initiative comprises a medical and a technical phase, seeking to compile lists of DCDEs and tackle harmonization and modeling issues, respectively. Although this paper remains at a conceptual level, it starts a discussion around the importance of DCDEs and launches a series of publications presenting the methods and findings of each planned phase. From early results, based on an ERN survey and a joint conference, DCDEs seem to be an essential extension to CDEs to increase interoperability, improve discoverability, and facilitate joint research collaborations. However, at this stage, ERN registries do not seem to have clear lists of DCDEs. The approach described for defining DCDEs appears to be feasible, as it shares many common steps with previous fruitful efforts on RD CDEs, as well as with others from outside the RD field. However, it remains dynamic and should be repeated regularly by curation teams, as DCDEs are expected to evolve over time based on arising research needs.

DCDE lists will be published, alongside CDEs, on the EU RD Platform in PDF format and added to ERDRI.mdr, the technical tool serving the purpose of a data dictionary. Semantic data modeling activities of the EJP RD, which currently focus on CDEs, can also be extended to DCDEs. The number of identified domains, as well as DCDEs per domain, should remain optimally minimal, as this eases their incorporation with CDEs in all new RD registries. However, in order to avoid differences in their interpretation and implementation across ERN registries, the EU RD Platform and EJP RD both have the role of raising greater awareness and encouraging the culture change necessary for their uptake and wide use.

## Appendix

Multimedia Appendix 1 List of the 24 initially funded European Reference Networks.

## Abbreviations

CDD: Common Data Dictionary
CDE: common data element
CRF: case report form
DCDE: domain-specific common data element
EJP RD: European Joint Programme on Rare Diseases
EPIRARE: European Platform for Rare Disease Registries
ERDRI: European Rare Disease Registry Infrastructure
ERN: European Reference Network
EU RD Platform: European Platform on Rare Disease Registration
EUCERD: European Union Committee of Experts on Rare Diseases Joint Action
JRC: Joint Research Centre
RD: rare disease
TF: task force
