# Supplementary material for: Domain-Specific Common Data Elements for Rare Disease Registration: Conceptual Approach of a European Joint Initiative Toward Semantic Interoperability in Rare Disease Research
Source: JMIR Med Inform. 2022 May 20;10(5):e32158. doi: 10.2196/32158 (PMC9166638; doi:10.2196/32158)
Supplement: Multimedia Appendix 1 [file medinform_v10i5e32158_app1.pdf]

List of the 24 initially funded ERNs

Acronym of the ERN	Name of the ERN
ERN BOND	European Reference Network on bone disorders
ERN CRANIO	European Reference Network on craniofacial anomalies and ear, nose and throat (ENT) disorders
Endo-ERN	European Reference Network on endocrine conditions
ERN EpiCARE	European Reference Network on epilepsies
ERKNet	European Reference Network on kidney diseases
ERN-RND	European Reference Network on neurological diseases
ERNICA	European Reference Network on inherited and congenital anomalies
ERN LUNG	European Reference Network on respiratory diseases
ERN Skin	European Reference Network on skin disorders
ERN EURACAN	European Reference Network on adult cancers (solid tumours)
ERN EuroBloodNet	European Reference Network on haematological diseases
ERN eUROGEN	European Reference Network on urogenital diseases and conditions
ERN EURO-NMD	European Reference Network on neuromuscular diseases
ERN EYE	European Reference Network on eye diseases
ERN GENTURIS	European Reference Network on genetic tumour risk syndromes
ERN GUARD-HEART	European Reference Network on diseases of the heart
ERN ITHACA	European Reference Network on congenital malformations and rare intellectual disability
MetabERN	European Reference Network on hereditary metabolic disorders
ERN PaedCan	European Reference Network on paediatric cancer (haemato-oncology)
ERN RARE-LIVER	European Reference Network on hepatological diseases
ERN ReCONNET	European Reference Network on connective tissue and musculoskeletal diseases
ERN RITA	European Reference Network on immunodeficiency, autoinflammatory and autoimmune diseases
ERN TRANSPLANT-CHILD	European Reference Network on Transplantation in Children
VASCERN	European Reference Network on Rare Multisystemic Vascular Diseases
